# *In Silico* Search of Energy Metabolism Inhibitors for Alternative Leishmaniasis Treatments

**DOI:** 10.1155/2015/965725

**Published:** 2015-03-30

**Authors:** Lourival A. Silva, Marina C. Vinaud, Ana Maria Castro, Pedro Vítor L. Cravo, José Clecildo B. Bezerra

**Affiliations:** ^1^Federal Institute of Education, Science and Technology of Goiânia, 76300000 Ceres, GO, Brazil; ^2^Goiás Network of Research in Biotechnology and Metabolomics of the Host-Parasite Relationship, Goiás Research Support Foundation (FAPEG), 74605050 Goiânia, GO, Brazil; ^3^Institute of Tropical Pathology and Public Health, Federal University of Goiás, 74605050 Goiânia, GO, Brazil

## Abstract

Leishmaniasis is a complex disease that affects mammals and is caused by approximately 20 distinct protozoa from the genus *Leishmania*. Leishmaniasis is an endemic disease that exerts a large socioeconomic impact on poor and developing countries. The current treatment for leishmaniasis is complex, expensive, and poorly efficacious. Thus, there is an urgent need to develop more selective, less expensive new drugs. The energy metabolism pathways of *Leishmania* include several interesting targets for specific inhibitors. In the present study, we sought to establish which energy metabolism enzymes in *Leishmania* could be targets for inhibitors that have already been approved for the treatment of other diseases. We were able to identify 94 genes and 93 *Leishmania* energy metabolism targets. Using each gene's designation as a search criterion in the TriTrypDB database, we located the predicted peptide sequences, which in turn were used to interrogate the DrugBank, Therapeutic Target Database (TTD), and PubChem databases. We identified 44 putative targets of which 11 are predicted to be amenable to inhibition by drugs which have already been approved for use in humans for 11 of these targets. We propose that these drugs should be experimentally tested and potentially used in the treatment of leishmaniasis.

## 1. Introduction

Leishmaniasis affects 12 million people worldwide, and approximately 350 million people from 98 countries are at risk of contracting the disease [1]. Leishmaniasis is caused by approximately 20 distinct species of* Leishmania* and is transmitted by two genera of phlebotomine sandflies:* Phlebotomus* in the Old World and* Lutzomyia* in the New World. From a clinical point of view, leishmaniasis is classified as cutaneous, mucocutaneous, or visceral; the latter is a severe form of the disease that becomes fatal if left untreated [1, 2]. As effective vaccines are unfortunately not available for either animals or humans, prevention is restricted to the combat of vectors, control of reservoirs, and treatment of affected individuals [3, 4].

The drugs currently approved for the treatment of leishmaniasis are directed at various molecular targets. Pentavalent antimonials interfere with the synthesis of DNA, *β*-oxidation of fatty acids, phosphorylation of ADP, and inhibition of glycolysis. Amphotericin B exhibits a high affinity for ergosterol, which is an important component of the cell membrane in fungi and* Leishmania.* Miltefosine induces apoptosis as a consequence of its intracellular accumulation. Although paromomycin inhibits cytochrome C in* Candida krusei*, its mechanism of action in* Leishmania* has not yet been elucidated; it is believed that its site of action is in the mitochondria, where it possibly interferes with the synthesis of proteins by hindering the translocation and recycling of ribosomal subunits. Pentamidine appears to reduce the membrane potential and inhibits the enzyme topoisomerase in the mitochondria [5]. However, all of these drugs exhibit serious problems, including drug resistance (antimonials), severe side effects (amphotericin and miltefosine), and a prohibitively high cost for use in a public healthcare setting (paromomycin and miltefosine) [6–8]. For these reasons, there is an urgent need for new drugs against leishmaniasis.

Although drugs may target lipids, nucleic acids, or polysaccharides, the drugs with the greatest efficacy are directed against protein targets [9]. The energy metabolism pathways of* Leishmania* include several protein targets that are interesting for the development or testing of new drugs [10, 11]. Glycosomes (similar to mammal peroxisomes) and mitochondria are the main sites of energy production in the promastigote forms of* Leishmania*. The glycosome is the site of glycolysis, which consists of seven reactions that break glucose down into two pyruvate molecules, while the mitochondria are the sites of the Krebs cycle and oxidative phosphorylation. In addition to glucose, amino acids are also important sources of energy. The final products of promastigote metabolism are carbon dioxide, succinate, pyruvate, D-lactate, alanine, ammonia, and urea [10]. The inhibition of one or more enzymes at each of these metabolic steps represents progress in the elimination of the parasite.

Prior to targeting a metabolic pathway, its importance for both the parasite and the host must be taken into consideration. The selectivity of inhibitors is also important, that is, how much they are able to inhibit a parasitic enzyme without causing any damage to the host [11]. Several enzymes in the* Leishmania* glycolytic pathway have been indicated as interesting therapeutic targets, including hexokinase, fructose-1,6-bisphosphate aldolase, triosephosphate isomerase, glyceraldehyde-3-phosphate dehydrogenase, phosphoglycerate kinase, pyruvate kinase, and glycerol-3-phosphate dehydrogenase [11]. However, the following question remains unanswered: which drugs inhibit these or other enzymes involved in the energy metabolism of* Leishmania*?

In general, the strategy for drug development includes both* de novo* discovery and the improvement of inhibitors of individually validated targets. Although this strategy is efficient for the development of new drugs against leishmaniasis, it is time-consuming and expensive. One interesting alternative approach is a strategy commonly known as drug repositioning. Drug repositioning makes use of known genomic data to search for drugs already approved for clinical use in humans for other diseases and for which the drug targets are already known. This approach uses the principle of “target homology” and can be put in practice using bioinformatics drug-target repositories, such as DrugBank [12] and Therapeutic Targets Database [13]. Such chemogenomics strategies considerably increase the likelihood of success in drug discovery, cutting down the costs and time spent in the process of research and development, in comparison with “traditional” methodologies. For this reason, pharma industries are increasingly using drug repositioning in order to find alternative applications for their drugs, reducing time and costs involved in the process of developing new compounds [14, 15].

In the present study, we used the concept of drug repositioning to identify new drugs with potential activity against* Leishmania* parasites. We first used genomic data to compile a list of energy metabolism drug targets in* Leishmania* of possible therapeutic interest. Each of these potential targets was then used as query in databases that include information on thousands of therapeutic compounds active against specific protein targets, the metabolic pathways that are involved, and the diseases for which they have been tested or for which clinical tests are currently in progress [12, 13, 16–19].

## 2. Materials and Methods

### 2.1. Compilation of a List of Potential* Leishmania* Energy Metabolism Targets

A list of hypothetic targets was extracted from the database TDR Targets (http://tdrtargets.org). TDR Targets is a project of the World Health Organization that prioritizes neglected tropical diseases. The database has open access and provides genomic information on specific species that allows users to identify and prioritize targets of interest [15, 20]. We selected* targets* on the first page, and in the first field, we selected* L. major* as the pathogenic species of interest. The next field provided a filter based on the following: Name/Annotation, Features, Structures, Expression, Antigenicity, Phylogenetic distribution, Essentiality, Validation data, Druggability, Assayability, and Bibliographic references. We decided to limit the number of filters to increase the odds that the search would produce results. Despite this, some of the genes related to* Leishmania* energy metabolism could not be found in the TDR Targets database. For this reason, we expanded the filter Name/Annotation and chose the option “Energy metabolism” in the field* KEGG high-level pathway.* Next, we expanded the filter* Druggability* and selected a druggability evidence range (which varies from zero to one) ≥0.2 in the option* Druggability index*. This query was automatically saved in the* my history* section; the results were organized into a table that could be uploaded.

### 2.2. Identification of Potential Drug Targets in Available Drug Databases: General Strategy

We ran searches of the name of each gene identified as TDR Targets in the database TriTrypDB (http://tritrypdb.org/tritrypdb/), which contains genomic information on pathogens of the family Trypanosomatidae, to which the genera* Leishmania* and* Trypanosoma* belong [21]. In the TriTrypDB homepage, we wrote the names of the genes in the field* Gene ID* and selected the* search* option. We checked each gene's name and product and extracted their predicted peptide sequences. In the cases of known enzymes whose encoding genes could not be identified among the TDR Targets, we wrote the enzyme name in the field* Gene Text Search* to run the search. Every* Leishmania* predicted peptide was considered as a potential target, and the sequences were used to run searches in the online databases DrugBank (http://www.drugbank.ca/) and Therapeutic Target Database (TTD) (http://bidd.nus.edu.sg/group/TTD/ttd.asp), which provide basic information on drugs and their primary or hypothetical targets.

DrugBank is an open-access database that provides users with information on drugs (chemical, pharmaceutical, and pharmacological data) and their targets (sequence, structure, and metabolic pathway). The database currently contains data on approximately 8,000 agents, including 1,700 drugs already approved by the FDA (Food and Drug Administration) and 6,000 experimental drugs [12, 17, 22]. The TTD stores information on 2,400 targets and approximately 21,000 drugs, of which 2,000 are already approved and 460 are currently undergoing clinical testing. In addition, it provides information on targets, such as sequence, function, metabolic pathway, and three-dimensional structure, through links to other relevant databases [13, 18, 19].

In both databases, our search strategy was based on the target-similarity principle, whereby each command (energy metabolism enzymes in* Leishmania*) was compared by similarity to all known drug targets included in all of the above-mentioned databases. Whenever homologous drug targets were identified, all of the proteins with an output expectation value (*E*-value) less than 1*E* − 5 relative to all three databases were included in the list of potential targets [14]. From this list, we selected the targets that interact with compounds that are already approved for clinical use in humans. Additional information on each drug was obtained from the PubChem database (https://pubchem.ncbi.nlm.nih.gov/).

### 2.3. Compilation of a List of Predicted Targets

Following the search of all of the enzymes involved in* Leishmania* energy metabolism in the two databases, all of the proteins for which the search results were negative were discarded. The remaining predicted targets from each database were compiled for the target spreadsheet provided by the TDR Targets. The following parameters associated with each positive hit were introduced in the spreadsheet: “Homologous target(s) name(s) and target ID(s)” (DrugBank and TTD), “*E*-value(s)” (DrugBank and TTD), “Drug type(s)” (DrugBank and TTD), “Drug name(s)” (DrugBank and TTD), “Drug ID(s)” (DrugBank), “Toxicity” (DrugBank), and Drug ID(s) and CID (PubChem).

### 2.4. List of Drugs for* In Vitro* Tests against* Leishmania* spp

In addition to the information gathered from the PubChem database, we performed a literature search of the PubMed database of approved drugs that have not yet been tested against* Leishmania*. We searched for all of the drugs associated with each positive hit on our list. Our definition of “test” included* in vivo* and/or* in vitro* tests and any species of* Leishmania*. Drugs were classified as “untested” when no related publication could be found. In these cases, one of the following search terms was introduced in PubMed: (1) (“drug name”[MeSH Terms] OR “drug name”[All Fields]) AND (“Leishmania”[-MeSH Terms] OR “Leishmania”[All Fields]) and (2) (“drug name”[MeSH Terms] OR “drug name”[All Fields]) AND (“Leishmaniasis”[MeSH Terms] OR “Leishmaniasis”[All Fields]). Drugs were also classified as “untested” when the information provided by the located study(ies) was insufficient to infer the potential usefulness of the drug as a leishmanicidal agent.

## 3. Results

### 3.1. Compilation of a List of Predicted Targets

**Figure 1:**
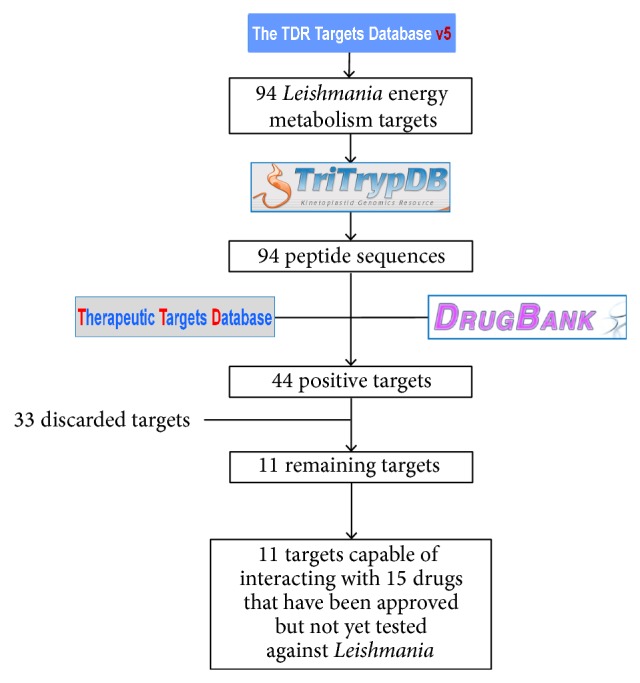
Flowchart depicting the overall strategy and results of this work.

To clarify the procedure we used for the identification and selection of the energy metabolism targets, all of the steps are depicted in Figure 1. We identified 94 genes associated with the energy of* Leishmania*. All of the products of these genes were considered as potential therapeutic targets. The predicted amino acid sequence of all these peptides was searched based on target similarity in the databases TTD and DrugBank.

A list of 44 positive hits (approximately 47% of the predicted peptides corresponding to* Leishmania* energy metabolism) was generated. Two databases were used to avoid missing important targets because the information they contained on drug targets was not the same (Figure 1). We identified 26 predicted targets in TTD, four of which appeared only in this database, and 40 predicted targets in the DrugBank database, 14 of which were exclusive to it. Twelve of the targets are enzymes that participate in glycolysis and/or the Krebs cycle, 13 participate in oxidative phosphorylation, and 11 participate in amino acid metabolism.

From the list of positive hits, we selected targets that had been previously tested in any organism and that had a corresponding commercially available drug. Detailed information on these targets and the corresponding compounds is provided in Table 1.

### 3.2. Untested Drugs

To establish which target-related drugs had been previously tested against leishmaniasis, we performed a literature search in the PubMed database, as described above. We found that only one of the target-related compounds had been previously tested against leishmaniasis.

## 4. Discussion

The aim of the present study was to identify drugs that are specifically active against* Leishmania* energy metabolism enzymes and have been previously tested against nonparasitic diseases or other human parasitic diseases. Below, we discuss the identified drugs and their effects on* Leishmania* energy metabolism.

Lonidamine (Doridamina) (LND, (1-[(2,4-dichlorophenyl)methyl]-1H-indazole-3-carboxylic acid) was developed as an antispermatogenic agent that proved to be effective against cells with high metabolic activity, such as cancer and protozoan cells [23, 24]. In tumor cells, LND impairs glycolysis and lactate production by inhibiting mitochondrial hexokinase [23]. This inhibition was achieved with a lethal dose (LD50) of ~260 *μ*M and ~80 *μ*M in* L. mexicana* promastigote forms and* Trypanosoma cruzi* epimastigote forms, respectively. The mechanism of action of LND was attributed to inhibition of the energy metabolism [23]. In* Trypanosoma brucei*, which causes African trypanosomiasis, LND inhibits the enzyme hexokinase in the procyclic forms of the parasite, indicating that hexokinase is a relevant therapeutic target for LND [25].

Nadide has been approved for the treatment of Parkinson's disease, Alzheimer's disease, and cardiovascular diseases (DrugBank data). Recent studies on breast cancer and longevity have revealed the importance of this small molecule. Nadide is the commercial form of the NADH molecule that living organisms produce through the reduction of nicotinamide adenine dinucleotide (NAD).

Appreciation of the importance of this small molecule has greatly increased since it was discovered at the beginning of the 20th century. The best-known role of NAD is as an electron carrier in oxidation-reduction reactions. However, several other functions have been attributed to it, including signal transduction and protein and DNA modification [26]. Recently, [27] reported that NAD+ homeostasis is crucial for* Leishmania* biology and virulence. The authors showed that nicotinamidase, which catalyzes the conversion of nicotinamide into nicotinic acid, is the key enzyme for the production of NAD+ from its precursors. Interestingly, the involved precursors (nicotinamide, nicotinic acid, and nicotinamide riboside) are derived from the host, and nicotinamidase cannot be found in mammals [27].

Sodium sulfacetamide or sulfacetamide is a bacteriostatic agent that is active against sulfonamide-sensitive Gram-negative and Gram-positive bacteria, including* Streptococci, Staphylococci, E. coli, Klebsiella pneumoniae, Pseudomonas pyocyanea, Salmonella spp.*,* Proteus vulgaris,* and* Nocardia*, which are usually isolated in secondary infections of the skin.

The LD50 of sulfacetamide for mice is 16,500 mg/kg by the oral route. In humans, the side effects include erythema, moderate swelling, nausea, vomiting, and headache. In addition to these side effects, the occurrence of Stevens-Johnson syndrome was reported in HIV-positive patients who received sulfacetamide drops for eye infections. All of these side effects, however, are associated with oral administration or high drug absorption through the skin, mucous membranes, and the conjunctiva, whereas topical use is not associated with strong side effects, except in the case of individuals with a hypersensitivity to sulfonamide.

Sulfacetamide inhibits mannose-6-phosphate isomerase (also known as phosphomannose isomerase (PMI)), which is considered the key enzyme in kinetoplastid energy metabolism. PMI catalyzes the isomerization of fructose-6-phosphate into mannose-6-phosphate. As the identity of the kinetoplastid PMI with human PMI is low (*E*-value = 3*E* − 59), it represents an interesting target on which to test new drugs.

## 5. Conclusions

We identified potential inhibitors of* Leishmania* energy metabolism by searching the information available in databases based on the target-similarity principle. Although approved for use in humans, none of the drugs identified have yet been tested against the parasite* Leishmania*.* In vitro* and* in vivo* tests may confirm the efficacy of these drugs, potentially leading to their use in the treatment of leishmaniasis.

## Figures and Tables

**Table 1:** New drug-target associations disclosed in the present study.

Drug (brand names)	Drug category(ies)	Toxicity	*Leishmania* target(s) ID	Identity to (*E*-value)	TDRT druggability
Lonidamine (Doridamina)	Anticancer, antitrypanosomal	NA	Hexokinase, putative LbrM.21.0310	Hexokinase D (3*E* − 54)	NA

POLYSIN	Antiprotozoal	NA	ATP-dependent phosphofructokinase LbrM.29.2480	Pyrophosphate-dependent phosphofructokinase (2.00*E* − 06)	NA

Saframycin A	Anticancer	NA	Glyceraldehyde 3-phosphate dehydrogenase LbrM.30.2950	Glyceraldehyde 3-phosphate dehydrogenase, muscle 2*E* − 91	NA

Nadide	Chagas disease, parasitic diseases, and sleeping sickness	No reports of overdose; however, high doses of NADH (10 mg a day or more) may cause jitteriness, anxiety, and insomnia	Glycerol-3-phosphate dehydrogenase LbrM.10.0640	Glycerol-3-phosphate dehydrogenase [NAD+], cytoplasmic 2*E* − 31	0.2

Sulfacetamide; sulfanilamide; sulfoxone	Bacterial infections	Oral LD50 mouse: 16500 mg/kg. Side effects include moderate to severe erythema (redness) and moderate edema (raised skin), nausea, vomiting, headache, dizziness, and tiredness. Higher exposure causes unconsciousness	Phosphomannose isomerase (PMI) LbrM.32.1750	Mannose-6-phosphate isomerase 3*E* − 59	0.6

Morantel tartrate, oxantel pamoate, and thiabendazole	*Helicobacter pylori *infection, Mature gastrointestinal nematode infections*, Trichuris trichiura *infection	Overdosage may be associated with transient disturbances of vision and psychic alterations	NADH-dependent fumarate reductase LbrM.34.0820	Fumarate reductase flavoprotein subunit 2.00*E* − 13	0.5

CEPHARANTHINE; CONESSINE	African trypanosomiasis; Chagas disease; *Leishmania* infections; parasitic diseases;	NA	Trypanothione reductase LmjF05.0350	Glutathione reductase, mitochondrial 6*E* − 65	NA

Aciglut, Glusate, Glutacid, Glutamicol, Glutamidex, Glutaminol, and Glutaton	Antibacterial; antitrypanosomal	Glutamate causes neuronal damage and eventual cell death, particularly when the NMDA receptors are activated. High dosages of glutamic acid may include symptoms such as headaches and neurological problems	Aspartate aminotransferase, putative LbrM.24.0370	Aspartate aminotransferase, mitochondrial 8*E* − 89	0.3

2-Oxopropanoic acid, pyroracemic acid, and acetylformic acid	Bacterial infections		Pyruvate kinase LbrM.34.0010	Pyruvate kinase 1.00*E* − 117	NA

Succinic acid (Katasuccin, Kyselina jantarova)	NA	Oral rat LD50: 2260 mg/kg	Succinate dehydrogenase LmjF15.0990	Succinate dehydrogenase [ubiquinone] iron-sulfur subunit, mitochondrial 2*E* − 28	NA

Adenovite, Cardiomone, Lycedan, My-B-Den, Phosaden, and Phosphaden	NA	NA	Acetyl-coenzyme A synthetase, putative LbrM.23.0580	Acetyl-coenzyme A synthetase, cytoplasmic 3*E* − 159	0.2

## Appendix

### Database Details and Commands

*DrugBank Database Commands*. In this database, the search was initiated by selecting the options “search” and then “Sequence Search” from the toolbar menu. The sequence of each protein investigated was entered in FASTA format in the appropriate field, and the search parameters were selected automatically.

*TTD Commands*. On the homepage of this database, we selected the option “target similarity search.” The sequence of the protein being investigated was entered in FASTA format in the appropriate field, and the option “search” was selected. In the cases with positive search results, only the targets with an *E*-value less than 1*E* − 5 were considered for further analysis.

*PubChem Database Commands*. In this database, the tab “compound” was selected, and every drug identified in DrugBank and TTD was entered in the appropriate field. The PubChem database was searched to gather detailed information on each compound, including the pharmacology, patents, physical and chemical properties, results of biological tests, biological interactions, and metabolic pathways.
